# Hear My Voice! The Experience of Self-Advocacy Among Patients with Enterostomy: A Qualitative Study

**DOI:** 10.3390/nursrep15090341

**Published:** 2025-09-19

**Authors:** Yanlin Shen, Yashi Zou, Juan Du, Shaoqi Chen, Jing Tan, Huijuan Ma

**Affiliations:** 1 School of Nursing, Army Medical University (Third Military Medical University), Chongqing 400038, China; lin18323386123@163.com (Y.S.); zysscii@163.com (Y.Z.); 19122305517@163.com (S.C.); 2 944th Hospital of the Joint Logistic Support Force of the Chinese People’s Liberation Army, Jiuquan 735000, China; 3 Department of Orthopedics, Xinqiao Hospital, Army Medical University (Third Military Medical University), Chongqing 400037, China; 4 Department of General Surgery, Daping Hospital, Army Medical University (Third Military Medical University), Chongqing 400042, China; dujuan@tmmu.edu.cn

**Keywords:** self-advocacy, patients with enterostomy, descriptive phenomenological method

## Abstract

**Objective:** This qualitative study aimed to understand the experience of self-advocacy among patients with enterostomy and to identify the behaviors, influencing factors, and benefits of self-advocacy. **Methods:** A descriptive phenomenological method was used, and the study was conducted from April to June 2024. A total of 17 patients with enterostomy were interviewed, and Colaizzi’s seven-step method was used to analyze interview data. **Results:** Three themes and thirteen sub-themes were generated: behaviors of self-advocacy (information seeking, effective communication, support seeking, and decision making), influencing factors of self-advocacy (type of enterostomy, economic burden, caregiving burden, stigma, and social support), and benefits of self-advocacy (enhancing self-care skills, enhancing physiological–psychological–social adaptability, dietary habit modification, and peer support). **Conclusions:** Self-advocacy is a critical tool for overcoming challenges, meeting needs, and cultivating connected strength, and targeted interventions could be designed to improve the levels of self-advocacy and self-management.

## 1. Introduction

As the third most common cancer worldwide, colorectal cancer (CRC) is predicted to occur in 3.2 million new cases and cause 1.6 million deaths annually by 2040 [1]. To minimize postoperative complications and preserve excretory function, enterostomy is performed to create a surgical opening in the abdominal wall after CRC surgery [2]. There are over 1 million enterostomy patients in China, with the number of cases increasing by more than 100,000 annually [3], and the number of enterostomy patients is estimated at 725,000 to 1 million in the United States [4]. Given the large number of patients with enterostomy worldwide, addressing the challenges they face is a major public health priority.

After discharge, patients with enterostomy face physiological, psychological, and social challenges. Stoma leakage is the most frequent physiological problem, and patients require education on stoma management [5,6]. A visible stoma, altered body image, reduced quality of life, and psychosocial adaptation may lead to anxiety and depression [5,6]. Because effective self-management is an important skill for patients with enterostomy, we developed a self-management framework based on King’s Conceptual System following a scoping review [5]. We also identified the interaction of information, communication, support, and decision-making with personal, interpersonal, and social systems [5]. These four interacting elements underpin the key behaviors of self-advocacy and stimulate our interest in exploring the experience of self-advocacy among patients with enterostomy.

As an effective means of encouraging healthier behaviors and improving health outcomes, self-advocacy empowers individuals to actively seek support, share their experiences, and raise awareness of cancer [7]. Since Clark and Stovall first applied the concept of self-advocacy among patients with cancer in 1996, experiences of self-advocacy have been explored in breast cancer, ovarian cancer, and lung cancer [8,9,10]. A qualitative meta-synthesis of self-advocacy experiences showed that patients with cancer may benefit from self-advocacy, including gaining confidence and improving self-management ability; they also encounter factors contributing to and hindering self-advocacy [11]. For patients with enterostomy, self-management requires dynamic interactions between personal, interpersonal, and social systems [5], with self-advocacy acting as the internal capacity to navigate these open systems. However, lived experiences of self-advocacy have not been explored among patients with enterostomy. Thus, this qualitative study aimed to understand these patients’ self-advocacy experiences, including clarifying the behaviors, influencing factors, and benefits of self-advocacy.

## 2. Materials and Methods

### 2.1. Design, Participants, and Setting

A qualitative study using a descriptive phenomenological method was conducted from April to June 2024. This design facilitates the exploration, analysis, and description of self-advocacy experiences among patients with enterostomy while maintaining the richness, breadth, and depth of this phenomenon to achieve more direct and primitive contact with their experiences [12]. The report followed the Consolidated Criteria for Reporting Qualitative Research (COREQ) checklist [13].

Purposive sampling was used to recruit patients with enterostomy in a tertiary hospital in Chongqing, China, and demographic and sociological information, including sex, age, living arrangement, living place, type of enterostomy, medical insurance, period after surgery, and complications of surgery, was considered to enrich the diversity of data when sampling. The inclusion criteria were as follows: (1) patients who had undergone enterostomy, (2) were over 18 years old, (3) could communicate, and (4) consented to participate. The exclusion criteria included (1) patients with a history of mental illness or existing mental disorders and (2) patients with advanced cancer and unstable condition. When no new topics emerged, group discussions on data saturation were held to establish consistency. When 15 participants were interviewed, saturation was reached. Another 2 participants were included to examine saturation, bringing the total to 17 patients in this study. This research was approved by the Ethics Committee of the Daping Hospital of the Army Medical University (No.124 of 2024), and written informed consent was obtained from each participant.

### 2.2. Data Collection

Semi-structured interviews were conducted to collect data. The outline of the interview included the following questions: (1) when did you undergo enterostomy surgery, and what did you experience at that time; (2) could you describe your current status of ostomy care, psychological well-being, dietary habits, and physical activity; (3) have you faced challenges, and how did you cope with them, (4) when faced with challenges, who did you seek support from; (5) have you shared ostomy care experiences with others?

The interviews were conducted in a quiet setting in a hospital office. Prior to the interviews, the researcher collected social demographic information from the participants and established trust with them. The purpose and significance of the interviews were explained to the patients, and they were assured that their confidentiality and willingness to participate would be respected. The patients were also asked for their consent to record the interviews, which lasted 30 to 40 min. Nonverbal information, such as facial expressions and body language, was observed and recorded. After the interviews, the audio files were transcribed into textual data and cross-checked by another researcher.

### 2.3. Data Analysis

This study used Colaizzi’s seven-step method to analyze interview data [14]. The steps were transcribing the descriptions of all the participants, extracting meaningful statements relating to their experiences of self-advocacy, coding meaningful statements, identifying and organizing the meaning units into theme clusters, merging the developed themes and describing them with detail, identifying the fundamental structure of the phenomenon, and returning the themes to participants for validation. Data coding and organization were conducted manually. Two researchers independently analyzed and coded the transcript, and a third researcher was invited to compare their results. When discrepancies occurred, group discussions were conducted with experts.

### 2.4. Quality and Rigor

Credibility, dependability, confirmability, and transferability were ensured to maintain data trustworthiness [15,16], and a team-based framework analysis was also conducted to achieve rigor. To achieve credibility, we spent extra time gaining an in-depth understanding of the participants’ experiences. To achieve dependability, we invited an external evaluator to review the research process. To achieve confirmability, we invited participants to verify the accuracy of the findings. To achieve transferability, we provided detailed descriptions of the findings.

In the meantime, the bracketing process with a reflective diary was conducted to manage potential research bias. Prior to recruitment and after the pilot interview, interviewers reflected on the pre-assumed response to each interview question and refined the questions as necessary. To minimize bias, none of the interviewers had pre-existing relationships with the participants. After each interview and when working with recordings and reading transcripts, the researchers also adopted reflective diary approaches to guide the bracketing process.

## 3. Results

**Figure 1:**
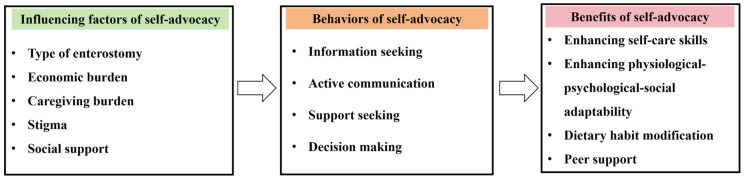
Themes and sub-themes.

A total of 17 patients with enterostomy were interviewed in this study. The mean age of the participants was 51.8 ± 15.37 years; seven (41.2%) were male and ten (58.8%) were female; five (29.4%) had temporary enterostomy and twelve (70.6%) had permanent enterostomy; and thirteen (76.5%) had surgical complications, while four (23.5%) did not experience complications. The participants’ sociodemographics are detailed in Table 1. The research findings included three themes and 13 sub-themes, as shown in Figure 1.

### 3.1. Behaviors of Self-Advocacy

Behaviors of self-advocacy among patients with enterostomy include information seeking, effective communication, support seeking, and decision making.

#### 3.1.1. Information Seeking

Patients with enterostomy could enhance their understanding of disease management by proactively seeking health information through multiple sources, which is one of the key behaviors of self-advocacy.

*The doctor mentioned earlier that I might need an ostomy. There were relevant materials in the ward, and some ostomy flyers posted in the hospital corridor. I also searched online for information–both from ward leaflets and online channels.*

(P15)

*After falling ill, I learned some medical knowledge by seeking information from various sources. *

(P04)

#### 3.1.2. Support Seeking

Patients with enterostomy could seek support to manage their ostomy bag. As one participant noted, ‘*You can’t manage to change the ostomy bag alone–it’s much easier with help. I slowly figured out how to do it*’ *(P03)*. These patients need support, especially when they are in a bad mood. As another participant noted, ‘*Radiotherapy and chemotherapy are not easy, but you must actively adjust. If you feel unhappy, you can vent to family, friends, or anyone you trust–always stay in a good mood*’ *(P15)*.

#### 3.1.3. Active Communication

Active communication serves as another behavior of self-advocacy, and patients can use communication to externalize psychological distress and solve problems related to enterostomy management.

*When I’m in a bad mood, I really like to talk to someone. *

(P14)

*When something happens, we ask the staff… We have never experienced this before, so when problems arise… We all consult the nurses. *

(P01)

#### 3.1.4. Decision Making

Patients initiate collaborative decision making by actively seeking professional guidance so their problems can be resolved.

*For any problems, we report them to doctors and nurses because we are not professionals. They are. *

(P09)

*I think the health care staff are great; they explain everything when I ask, and any problems can be solved. *

(P11)

### 3.2. Theme 2: Influencing Factors of Self-Advocacy

Influencing factors of self-advocacy among patients with enterostomy include type of enterostomy, economic burden, caregiving burden, stigma, and social support.

#### 3.2.1. Type of Enterostomy

Patients with temporary enterostomy exhibited reduced disease-related apprehension. As one participant noted, *‘Since it’s temporary, I didn’t bother to understand much—after all, it will be reversed. It’s not permanent... There’s nothing to worry about’ (P07)*.

#### 3.2.2. Economic Burden

Enterostomy management materials would be an economic burden for some patients, which could affect self-advocacy.

*The price of ostomy bags is too high for us to afford. *

(P16)

#### 3.2.3. Caregiving Burden

Patients with a heavy burden of care may neglect their own health. As one participant noted, *‘The two of us live together. But my spouse has three conditions: diabetes, Parkinson’s disease, and epilepsy—a neurological disorder. Epilepsy is the most troublesome; she can’t cook now, so I end up taking care of her instead’ (P02)*.

#### 3.2.4. Stigma

Stigma surrounding ostomy visibility may limit their self-advocacy.

*I feel uncomfortable wearing an ostomy bag, worried about others discovering it. I think it would be embarrassing if others knew. *

(P16)

#### 3.2.5. Social Support

Social support from family members and professionals is a key contributing factor of self-advocacy.

*I can’t manage it myself, so my son usually changes the ostomy bag. I still rely on him a lot. *

(P06)

*My niece helps me change it... Honestly, my family manages it very well. *

(P11)

*I think the healthcare staff are great; they explain everything when I ask, and any problems can be solved. *

(P11)

### 3.3. Theme 3: Benefits of Self-Advocacy

Benefits of self-advocacy among patients with enterostomy include enhancing self-care skills, enhancing physiological–psychological–social adaptability, modifying dietary habits, and receiving peer support.

#### 3.3.1. Enhancing Self-Care Skills

Participants developed the ability to manage input compilations, such as leaks, through experiential learning and resourceful adaptation of solutions.

*The base plate had a rigid ring, so when pressed, it would leak several times... I learned from experience how to deal with leaks. *

(P02)

*I explored and found a skin patch material similar to that used for babies. *

(P10)

*I manage and handle the ostomy more skillfully now. *

(P17)

#### 3.3.2. Enhancing Physiological–Psychological–Social Adaptability

By actively maintaining exercise and social hobbies, participants enhanced their physiological, psychological, and social adaptability.

*My activities are not affected. Thankfully, I’m still young. In my spare time, I can go fishing, something I enjoy. I still go fishing every day to keep up with my hobbies. *

(P01)

*I hang out with other people. When I sing, I’m happy. I love singing. *

(P11)

*Changing lifestyle habits was difficult at first, but after hearing other people’s advice, I thought the more I take care of my body, the healthier it will be. I stopped feeling anxious and gradually accepted the situation. *

(P10)

#### 3.3.3. Dietary Habit Modification

After enterostomy, patients need to adjust dietary habits to adapt to physiological changes by avoiding gas-producing food.

*I can eat normally, except for trigger foods and gas-producing items. *

(P01)

*I follow the doctor’s dietary instructions. *

(P04)

*I’ve become more attentive to what I eat now. *

(P17)

#### 3.3.4. Peer Support

Peer support emerged as one of the benefits of self-advocacy, and experienced patients could help others overcome practical challenges. As one participant noted, *‘We communicate in our WeChat group. I once helped a fellow patient complete special disease insurance procedures earlier than expected, so I quickly shared the experience with others’ (P06)*.

## 4. Discussion

This qualitative study identified self-advocacy, including the behaviors, influencing factors, and benefits associated with it, as a dynamic process in patients with enterostomy. Information seeking, effective communication, support seeking, and decision making are interconnected self-advocacy behaviors that are consistent with the four interacting elements in the patient self-management framework of patients with enterostomy developed by our research team [5]. Thomas et al. (2021) identified informed decision making, effective communication with healthcare providers, and connected strength as specific self-advocacy behaviors among women with cancer [17]. Self-advocacy behaviors of patients with enterostomy address interconnected physical, psychological, and social needs arising from stoma management [18].

Patients with enterostomy encounter factors contributing to and hindering self-advocacy, including type of enterostomy, economic burden, caregiving burden, stigma, and social support. Stoma-related economic burden [19] could also be a major hindering factor of self-advocacy among patients with enterostomy. Past research has shown that more than half of the caregivers of patients with cancer have a high level of caregiving burden [20], revealing that this factor should also be considered. Due to stoma and stigma, CRC survivors with permanent enterostomy are reported to have moderate to high social isolation [21], especially during the discharge transition period [22]. Tailored interventions could be conducted to improve stoma acceptance and reduce stigma [21], and these severe negative emotions are also closely related to social support levels [22].

Patients with enterostomy benefit from self-advocacy by gaining enhanced self-care skills, which are instrumental for disease adaptation once discharged [23]. Past research has shown that multimedia patient education interventions are more effective at providing self-care knowledge and skills than conventional instruction [23,24]. Dietary modification is paramount for reducing stoma output and preventing complications among patients with enterostomy [25], and our study reveals that self-advocacy also benefits successful dietary adaptation. Specifically, self-advocacy transforms dietary adaptation from passive compliance to proactive improvement.

Past research has shown that the psychosocial adjustment level of patients with enterostomy is at the lower end of the median range [2], and our study revealed that self-advocacy benefits psychosocial adjustment. Self-advocacy empowers patients with cancer to work effectively with healthcare providers to ensure that their health priorities are met [11]. This connected strength not only supports patients but also generates the energy to support peers through experience-sharing and raising awareness about cancer [17].

This qualitative study provides a new perspective for understanding patients with enterostomy; self-advocacy represents a philosophical approach in which patients can actively participate in healthcare interactions and improve their health outcomes [26]. However, this study has several limitations. The findings were generated from patients with enterostomy in China, so context-dependent findings cannot be generalized to all patients. The participants’ statements were translated from Chinese to English with the assistance of a professional English editor to ensure accuracy. Back translation was then conducted by a professional translator, and the translation was reviewed by the researchers.

## 5. Conclusions

This qualitative study involved interviewing 17 patients with enterostomy to explore their experiences of self-advocacy. We generated three themes and thirteen subthemes. The findings demonstrate that self-advocacy is a critical tool for overcoming challenges, meeting needs, and building connected strength. The findings also demonstrate the need to improve and integrate self-advocacy into the discharge protocol. Self-advocacy skills are positively correlated with transition readiness [27], and nurses can use the self-advocacy scale to assess patients’ self-advocacy status and design education manuals to facilitate patient-centered care [28]. Due to the individualized self-advocacy of patients with cancer [29], future studies could be conducted to design targeted interventions to improve the level of self-advocacy and self-management. When designing interventions, influencing factors of self-advocacy can be used to find potential targets, and skills in information seeking, effective communication, support seeking, and decision making can be emphasized.

## Figures and Tables

**Table 1:** The sociodemographic characteristics.

Category		Number	Percentage
sex	male	7	41.2
	female	10	58.8
living arrangement	with children and spouse	8	47.0
	with spouse	7	41.2
	live alone	1	5.9
	with parents	1	5.9
living place	city	14	82.4
	countryside	3	17.6
type of enterostomy	permanent	12	70.6
	temporary	5	29.4
medical insurance	employee medical insurance	13	76.5
	commercial insurance	3	17.6
	resident insurance	1	5.9
period after surgery	1–3 month	6	35.3
	4–6 month	2	11.8
	7–12 month	3	17.6
	over 1 year	6	35.3
complications of surgery	yes	13	76.5
	no	4	23.5

## Data Availability

The data supporting the findings of this study are available upon request from the corresponding author.

## Public Involvement Statement

No patients or members of the public were involved in any aspect of this research.

## Guidelines and Standards Statement

This manuscript was drafted in accordance with the Consolidated Criteria for Reporting Qualitative Research (COREQ).

## Abbreviations

The following abbreviations are used in this manuscript:

COREQ	Consolidated Criteria for Reporting Qualitative Research
CRC	Colorectal Cancer
